# Report on Leprosy and the Trinidad Leper Asylum for 1892

**Published:** 1893-12-30

**Authors:** Beaven Rake

**Affiliations:** Trinidad


### THE INSTITUTIONAL WORKSHOP.

### REPORT ON LEPROSY AND THE TRINIDAD LEPER ASYLUM FOR 1892.

By BEAVEN RAKE, M.D.Lond. (Trinidad, 1893.)

This is a very interesting report, by an accomplished physician, on what appears to be, on the whole, a very well-managed leper hospital. That the unhappy inmates are kindly treated, and that their lives are rendered less miserable, is best proved by the facts that discharge from the asylum is employed as a punishment, and that the importunity with which those discharged seek readmission shows that such dismissal is the greatest punishment possible. Both men and women are employed, so far as is practicable, either in gardening or in sewing; the results are decidedly economical, and "there is no doubt that suitable employment is a most important factor, not only in preventing disorderly conduct, but also in the treatment of the disease." The number of inmates averages over 200, and the annual mortality is about 11 per cent., the cause of death in the majority being set down either as tuberculosis or kidney disease.

On the question of the communicability of leprosy, Dr. Rake abstains from dogmatising. "There are probably few bacteriologists," he says, "who would deny the possibility of leprosy being communicated; but the matter appears in a very different light when viewed from a practical standpoint." The author is by no means an alarmist on the subject of the alleged increase of leprosy. In 1815, it seems, Sir Ralph Woodford, then Governor of Trinidad, ascertained the number of lepers in the island to be 77. In 1890 this number had grown to 414, of whom 210 were inmates of the leper asylum. But, says Dr. Rake, the population of the island had meantime increased from 32,000 to about 200,000; wherefore the relative number of lepers was actually smaller, though the absolute number had grown enormously.

In Norway the leper population decreased from 2,900 to 1,195 during the thirty years, 1856-85, and concurrently with a considerable increase in the general population of the country. Yet until 1885 there was no law compelling the isolation of lepers. In India there was an absolute decrease, according to the Governmental census, between 1881 and 1891, although some districts were not included in the enumeration of the former year. But the most hopeful of all the facts quoted in this report is this: that in certain of the north-western (or more properly, north-central) States of the American Union, into which there has been for many years a large Norwegian immigration, but in which leprosy is not endemic, the disease "is strictly limited to the immigrants, and has never appeared in the descendants of lepers, nor in any one born in the State (Minnesota)."

As regards morbid anatomy, the three forms of leprosy were tolerably equally represented in Dr. Rake's 109 autopsies, the anæsthetic, however, being rather the most prevalent, and the mixed the least so. Dr. Rake considers the mixed form to be almost always a late development of the tuberculated. Here, as elsewhere, the duration of life in the anæsthetic form is greatest. Visceral lesions are found, post mortem, to be "almost, if not quite as common in anæsthetic as in tuberculated leprosy"; but are probably developed at a later period in the milder form.

Dr. Rake's figures certainly indicate that in Trinidad the anæsthetic form belongs, as a rule, to a more advanced age than the tuberculated; also that the mixed form is usually developed from the tuberculated cases of somewhat late origin, while those which begin early are apt to prove fatal in a few years, without allowing time, as it were, for the secondary anæsthetic development which would bring them into the mixed type. Thus the average age at death in the different types is widely discrepant. Dr. Rake, with a caution as to the uncertainty about their ages which prevails among his dark-skinned *clientele*, puts the average death-age in the tuberculated at 24, in the mixed type at 40, and in the anæsthetic at 44 years.

It is a point of great interest that whereas Dr. Rake examined microscopically the kidneys in the great majority of his 109 cases, and whereas various kidney lesions were found in 35 of these, in not one of the 35 was a leprosy bacillus met with. On the other hand, in seven of those cases where the kidney appeared healthy to the naked eye leprosy bacilli were discovered. These observations agree with those of Cornil and Babes, who describe albuminous nephritis as coming on, with lardaceous changes in other viscera, as the result of ulcerations of the skin and mucous membranes, but state that the bacillus lepræ may invade the kidneys and all the tissues of the body without producing any naked-eye change.

Another interesting point is that lepers as a rule bear well the results of surgical operations, incisions "usually healing with astonishing rapidity, producing firm cicatrices as quickly, if not more so, than in non-leprous patients." This seems to be due to the abundance of fibrine and the very rapid clotting which takes place in the blood of lepers. Dr. Rake seems to have taken full advantage of these peculiarities—his list of operations is an extremely long one, amounting to about 2,000 in six years, including 117 amputations, 102 nerve-stretchings, and 36 removals of tubercles. This last operation is often of great though temporary service; recurrence does not take place in the cicatrix.

The arduous work of nursing in the hospital has for 25 years been performed by a body of Dominican nuns. JOHN BEDDOE, M.D., LL.D., F.R.S.

